# Relationships between screen time and childhood attention deficit hyperactivity disorder: a Mendelian randomization study

**DOI:** 10.3389/fpsyt.2024.1441191

**Published:** 2024-09-23

**Authors:** Zhuo Meng, Bo Ao, Wei Wang, Tongtong Niu, Yanan Chen, Xiaoqing Ma, Youliang Huang

**Affiliations:** ^1^ School of Management, Beijing University of Chinese Medicine, Beijing, China; ^2^ Department of Pediatrics, China-Japan Friendship Hospital, Beijing, China; ^3^ National Institute of Chinese Medicine Development and Strategy, Beijing University of Chinese Medicine, Beijing, China

**Keywords:** childhood attention deficit hyperactivity disorder, Mendelian randomization study, screen time, causal relationships, genetic

## Abstract

**Background:**

In previous observational studies and meta-analyses, childhood attention deficit hyperactivity disorder (ADHD) is found to have a significant association with screen time. However, the causal associations between them remain unclear.

**Method:**

This study performed a bidirectional two-sample Mendelian randomization (MR) analysis to confirm the causality between screen time and childhood ADHD. Large-scale genome-wide association studies (GWAS) datasets derived from the Psychiatric Genomics Consortium (PGC) and the UK Biobank were used to identify single nucleotide polymorphisms (SNPs) associated with exposure and outcome. Four categories of datasets were selected to represent screen time. The SNPs that are significantly associated with exposure data (P < 5e-08) and have a strong correlation with the exposure in the F-statistic (F > 10) were selected as instrumental variables. This study also used the PhenoScanner V2 database and the LDlink webtool to exclude confounding factors, and the MR-PRESSO method (p < 0.05) was employed to eliminate outliers with bias. Five commonly used methods were employed to assess the interaction and the Inverse Variance Weighted (IVW) method was utilized as the primary basis for determining the MR estimates in this study.

**Results:**

The MR analysis revealed that the length of mobile phone use (OR, 1.848; 95% CI, 1.3360-2.5558; p=2.07e-4) and the time spent watching television (OR, 2.104; 95% CI, 1.3958-3.1703; p=3.8e-4) increased the risk of childhood ADHD. Although the causal relationships were exclusively identified through the IVW and weighted median methods, the results retained their statistical significance following correction. In the reverse analysis, no evidence was found to support an effect of childhood ADHD on screen time. The sensitivity analysis conducted on the significant findings revealed no evidence of horizontal pleiotropy or heterogeneity.

**Conclusion:**

This study provides some evidence for the causality of screen time and childhood ADHD. Given the limitations of our study, further research is required to comprehensively investigate this relationship.

## 1 Introduction

Attention Deficit Hyperactivity Disorder (ADHD) is a long-term neurodevelopmental condition, with predisposing factors occurring in early childhood (1), whose manifestations and affective effects can last into adulthood (1). Approximately 8–12% of children worldwide are diagnosed with ADHD (2), and over 60% of them experience psychological disorders and symptoms well into adulthood (3, 4). According to the results of a recent meta-analysis, in the world, the prevalence of childhood ADHD is approximated to be 7.2% (5), while the disease affects about 2.5% of adults (6). The Global Burden of Disease (GBD) study indicates that between 1990 and 2019, the global age-standardized incidence and prevalence of ADHD declined by -4.77% and -8.75%, respectively (7). However, the prevalence and disease impact of ADHD may be underreported according to the GBD figures (7). An increasing number of children with ADHD causes heavy burdens on society, families, and individual development. It is well known that the etiology of ADHD involves a complex interplay between environmental and genetic risk factors. Several studies have demonstrated that psychosocial disorders, environmental pollutants, and prenatal and postnatal variables are prevalent risk factors for ADHD. However, the origin and pathophysiology of ADHD are still unclear due to the disorder’s complexity and variety.

There is growing concern that youngsters who spend too much time on screens may develop psychological and cognitive issues as a result of their excessive screen usage. The suggested daily limit of two hours can be readily exceeded by children due to their lack of self-control. This average daily screen time for 8- to 18-year-olds increased by 1.17 hours in the ten years between 1999 and 2009 (8). The proportion of children aged 0 to 8 who had access to a mobile device increased, rising from 52% in 2011 to 75% in 2013 (9).

However, there is still a debate over the intrinsic link between screen usage and childhood ADHD. Reviews have indicated a strong association between screen exposure and ADHD or characteristics linked with ADHD, such as impulsivity and inattention (10, 11). A cross-sectional study from the Karachi National Institute of Child Health demonstrated a positive correlation between screen usage and ADHD in children (12). Nevertheless, research from the tertiary care facility in Sri Lanka found no correlation between screen time and clinically diagnosed ADHD (13).

Mendelian randomization (MR) is an analytical approach for ascertaining the causality between exposure variables and outcomes. As instrumental variables (IVs), the method makes use of single nucleotide polymorphisms (SNPs) derived from pooled data in genome-wide association studies (GWAS). If the SNP associated with the genetic variant is specifically linked to the exposure factor and impacts the disease solely through the exposure factor, then exposure factors are deemed causally related to disease. Since MR is based on the theory that genetic variants are randomly assigned during transmission from parent to child, it can mitigate the effects of reverse causality and other confounding variables by satisfying specific conditions. This means that when it comes to establishing the causal association between environmental factors and disease, MR is able to offer greater evidence than traditional observational research. Therefore, this study sought to investigate the possible causality between screen time and childhood ADHD using bidirectional two-sample MR analysis and to offer fresh data supporting the need for early intervention and treatment of ADHD.

## 2 Methods

### 2.1 Study design

**Figure 1:**
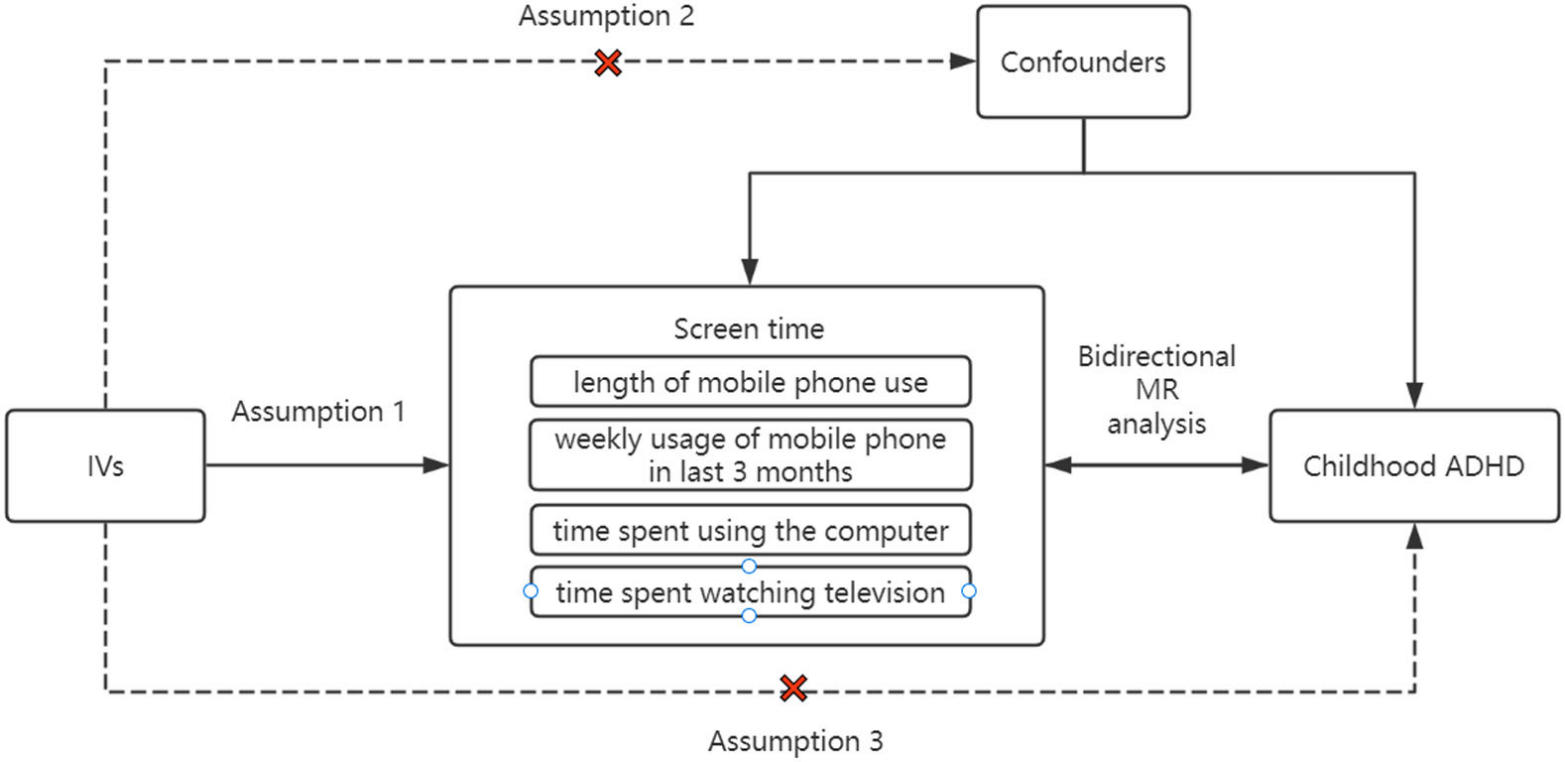
Three core assumptions of MR analysis in this study.

This study employed bidirectional two-sample MR analysis to confirm the causality between screen time and childhood ADHD. Four categories of datasets representing screen time were chosen, including time spent watching television, length of mobile phone use, time spent using the computer (Qu et al.)^1^, and weekly usage of mobile phone in last 3 months (14, 15). Based on the GWAS database, the data collected from European populations, as with the ADHD dataset. Initially, we used the data on screen time in four categories as exposure and the ADHD data as outcome. Afterwards, we switched the screen time data and ADHD data for MR analysis. Throughout the Mendelian randomization analysis, the following three assumptions had to be strictly met (**Figure 1**): (1) the close association between exposure and instrumental variables; (2) the independence of instrumental variables from any potential confounders that may affect the results; and (3) the exclusive impact of instrumental variables on outcomes through exposure, without any influence from other factors.

### 2.2 Data source for screen time and ADHD

The GWAS summary data for ADHD was obtained from the Psychiatric Genomics Consortium (PGC) (https://pgc.unc.edu/for-researchers/download-results), consisting of 225,534 individuals (186,843 healthy controls and 38,691 ADHD patients). The diagnosis of ADHD in all cases was conducted by psychiatrists following ICD10 (F90.0, F90.1, F98.8), which resulted in the identification of 27 genome-wide loci and 76 potential risk genes independently associated with ADHD (16).

The screen time data came from self-reported questionnaires collected by the UK Biobank Assessment Center (ACE). The questionnaires included data from eight sub-surveys on physical activity, electronic device use, insomnia, and smoking. The electronic device usage survey contained the use of cell phones (time of use, side of the head) and computer games. Two datasets derived from this survey were selected for the study: length of mobile phone use and weekly usage of mobile phone over past 3 months. In addition, data on time spent using the computer and time spent watching television were added to the exposure data to increase the breadth of the study. All exposure data were from European populations and consisted of four datasets (ukb-b: 5192, 4094, 4522, 17999). The dataset ukb-b-5192 included 437887 participants; dataset ukb-b-4094 included 456972 participants; dataset ukb-b-4522 included 360895 participants; dataset ukb-b-17999 included 386626 participants. All the data sources used for MR analysis are detailed in **Table 1**.

**Table 1:** Overview of the GWAS summary statistics.

GWAS ID/Source	Trail	Consortium	Population	Build
the PGC website	ADHD	PGC	European	
ukb-b-4094	Length of mobile phone use	MRC-IEU	European	HG19/GRCh37
ukb-b-17999	Weekly usage of mobile phone in last 3 months	MRC-IEU	European	HG19/GRCh37
ukb-b-4522	Time spent using computer	MRC-IEU	European	HG19/GRCh37
ukb-b-5192	Time spent watching television (TV)	MRC-IEU	European	HG19/GRCh37

### 2.3 Instrumental variables selection

This study extracted genome-wide SNPs that are significantly associated with exposure data (P < 5e-08) as initial instrumental variables. Using a reference panel of 1,000 genomic European populations, SNPs with linkage disequilibrium (LD) in the initial instrumental variables were removed under the threshold condition (R2 < 0.001) and a window size of 10 Mb to ensure the independent validity of the SNPs. Subsequently, the formula F = β^2^/se^2^ was used to calculate the F-statistic for each SNP and screen for SNPs with a strong correlation with the exposure data (F > 10). To exclude the influence of confounding factors on causal effects, we retrieved the relevant traits of strongly correlated SNPs from the PhenoScanner V2 database and the LDlink webtool, including negative mood, educational status, body mass index, ADHD behavior, mental disorders and other diseases. Outliers with bias in the SNPs were screened and eliminated using the MR-PRESSO method (p<0.05). The retained SNPs were used as final instrumental variables for conducting Mendelian randomization analysis.

### 2.4 Bidirectional analyses of Mendelian randomization

To assess the potential causal association between screen time and childhood ADHD, a two-sample MR analysis was conducted using four distinct categories of screen time as exposure factors and ADHD as the outcome variable. Five common methods were used to assess the interaction: inverse variance weighting (IVW), weighted median, MR-Egger, weighted, and simple median. Compared to other methods, the IVW method provides more accurate and stable assessment results (17, 18). Therefore, the IVW method was utilized as the main basis for determining the MR estimates in this study. Reverse Mendelian randomization analyses were conducted to test the possibility of reverse causality, using ADHD as the exposure factor and the four categories of screen time as the outcome variable.

### 2.5 Sensitivity analysis

In this study, the MR-Egger regression intercept method was applied to evaluate for horizontal pleiotropy in the MR estimates, with a p-value < 0.05 considered to be horizontal pleiotropy. Horizontal pleiotropy indicates that genetic variants influence multiple biological pathways, which contradicts the fundamental assumption of IVs in MR analysis. Specifically, IVs are presumed to affect the outcome exclusively through the exposure of interest. A p-value of less than 0.05 in the horizontal pleiotropy test suggests that the IVs employed in the MR analysis violate this premise. Consequently, the conclusions derived from the analysis lack statistical significance. The Cochran’s Q statistic was utilized to test for heterogeneity using the IVW method and the MR-Egger regression method, with a p-value < 0.05 considered to be heterogeneous. Leave-one-out sensitivity analyses were applied to assess the presence of directional pleiotropy and determine the effect of each SNP on the causal effect. MR-PRESSO tests were used to identify outliers in the SNPs, and MR analyses were performed again after removing the outliers. The entire analysis was carried out using RStudio (version 4.2.1) with the packages “MendelianRandomization”, “TwoSampleMR”, and “VariantAnnotation”.

## 3 Results

### 3.1 Selection of IVs

This study initially identified 32, 6, 82, and 112 valid SNPs as instrumental variables for the length of mobile phone use, weekly usage of mobile phones in the last 3 months, time spent using the computer, and time spent watching television. All of these SNPs were independent in LD and had F-statistics greater than 10. The number of excluded SNPs that contained traits such as negative mood, educational status, body mass index, ADHD behavior, psychiatric disorders, and other disorders were 15, 4, 46, and 76 for four types of screen time, respectively.MR-PRESSO outlier detection removed 2, 0, 0, and 2 outliers. 13, 1, 34, and 33 were the final numbers of instrumental variables employed in the MR analysis. The GWAS data of weekly usage of mobile phone over the past 3 months was screened with only one SNP for MR analysis, preventing the use of analysis methods such as IVW. Therefore, only the results of the other three types were considered.

### 3.2 Causal effect of screen time on childhood ADHD

**Figure 2:**
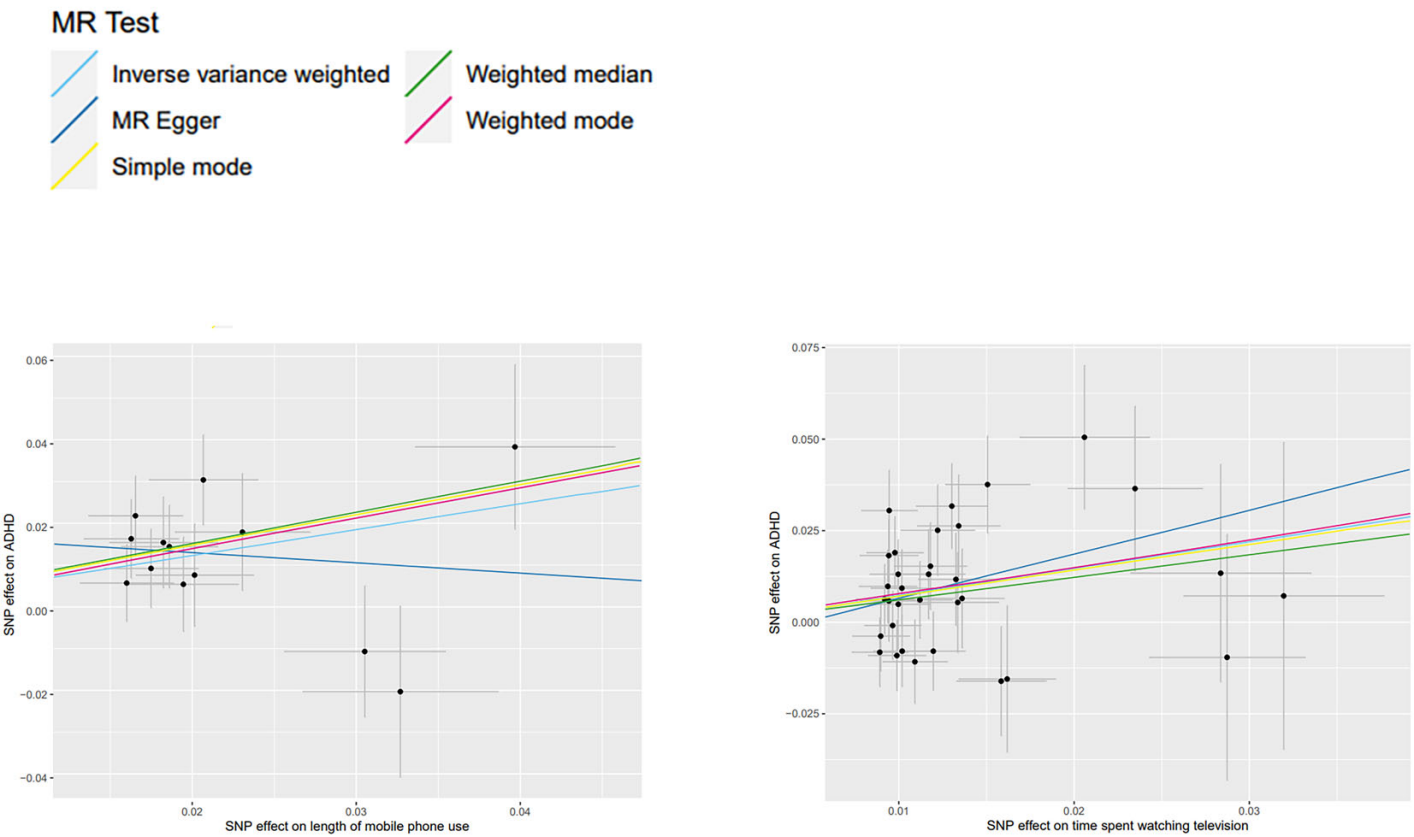
Two scatter diagrams of MR analysis. Exposure two screen times and outcome ADHD.

The IVW method revealed that the length of mobile phone use (OR, 1.848; 95% CI, 1.3360-2.5558; p=2.07e-4), the time spent watching television (OR, 2.104; 95% CI, 1.3958-3.1703; p=3.8e-4) had a causal relationship with childhood ADHD. To enhance the reliability of the results, we conducted a False Discovery Rate (FDR) correction employing the Benjamini-Hochberg method to mitigate the impacts of multiple exposures on our findings. The adjusted p-value remained below 0.05 (**Table 2**), indicating that prolonged exposure to both categories of screen time is associated with an elevated risk of childhood ADHD. Before and after adjustment, the Weighted median method consistently produced results consistent with the IVW method, while the other methods exhibited statistically insignificant p-values. Additionally, no significant association was found between the time spent with computers (IVW: OR, 0.719; 95% CI, 0.4818-1.0716; p=0.105) and childhood ADHD in any of the MR methods. Detailed statistical results of five models are provided (**Table 2**). After rigorous exclusion of SNPs, our study provides evidence that screen time and childhood ADHD may be causally related (**Figure 2**).

**Table 2:** Causal effects of three screen times on ADHD.

Exposure	Outcome	No. of SNPs	Method	Odds Ratio[95%CI]	P-value	P-adj
Length of mobile phone use	ADHD	13	MR Egger	0.782[0.2165, 2.8261]	0.715	
			Weighted median	2.104[1.3480, 3.2840]	0.001	0.003
			IVW	1.848[1.3360, 2.5558]	2.07e-4	5.7e-4
			Simple mode	2.081[0.9660, 4.4848]	0.086	
			Weighted mode	2.081[0.9819, 4.4121]	0.080	
Time spent using computer	ADHD	34	MR Egger	1.466[0.1889, 11.3692]	0.717	
			Weighted median	0.794[0.4887, 1.2900]	0.351	0.351
			IVW	0.719[0.4818, 1.0716]	0.105	0.105
			Simple mode	1.402[0.4185, 4.6984]	0.587	
			Weighted mode	1.424[0.4634, 4.3776]	0.541	
Time spent watching television	ADHD	33	MR Egger	3.346[0.6820, 16.4117]	0.147	
			Weighted median	1.850[1.0834, 3.1575]	0.024	0.036
			IVW	2.104[1.3958, 3.1703]	3.8e-4	5.7e-4
			Simple mode	2.025[0.5378, 7.6255]	0.305	
			Weighted mode	2.115[0.5240, 8.5408]	0.301	

### 3.3 Causal effect of childhood ADHD on screen time

This study performed MR analyses utilizing ADHD data as the exposure and screen time as the outcome to elucidate the inverse associations. The results (**Table 3**) indicated that there is minimal evidence to suggest a reverse causality between screen time and childhood ADHD. All of the IVW results were not statistically significant. A weak causal relationship was identified exclusively in the findings derived from the Weighted Median method between childhood ADHD and both the weekly mobile phone usage over the past 3 months (OR, 1.037; 95% CI, 1.0013-1.1169; p=0.045) and the time spent watching television (OR, 1.039; 95% CI, 1.0038-1.0758; p=0.030). However, their corrected p-value was greater than 0.05.

**Table 3:** Causal effects of ADHD on three screen times.

Exposure	Outcome	No. of SNPs	Method	Odds Ratio[95%CI]	P-value	P-adj
ADHD	Length of mobile phone use	4	MR Egger	1.026[0.9110, 1.1548]	0.716	
			Weighted median	1.043[0.9840, 1.1047	0.158	0.237
			IVW	1.040[0.9912, 1.0917]	0.109	
			Simple mode	1.048[0.9764, 1.1243]	0.285	
			Weighted mode	1.048[0.9716, 1.1300]	0.312	
ADHD	Time spent using computer	4	MR Egger	1.036[0.9345, 1.1479]	0.552	
			Weighted median	0.990[0.9538, 1.0282]	0.612	0.612
			IVW	0.993[0.9523, 1.0270]	0.565	
			Simple mode	0.993[0.9014, 1.0281]	0.321	
			Weighted mode	0.993[0.9313, 1.0522]	0.761	
ADHD	Time spent watching television	4	MR Egger	1.022[0.9211, 1.1331]	0.713	
			Weighted median	1.039[1.0038, 1.0758]	0.030	0.090
			IVW	1.034[0.9994, 1.0693]	0.054	
			Simple mode	1.056[1.0010, 1.1137]	0.116	
			Weighted mode	1.056[0.9893, 1.1270]	0.177	

### 3.4 Sensitivity analysis

**Figure 3:**
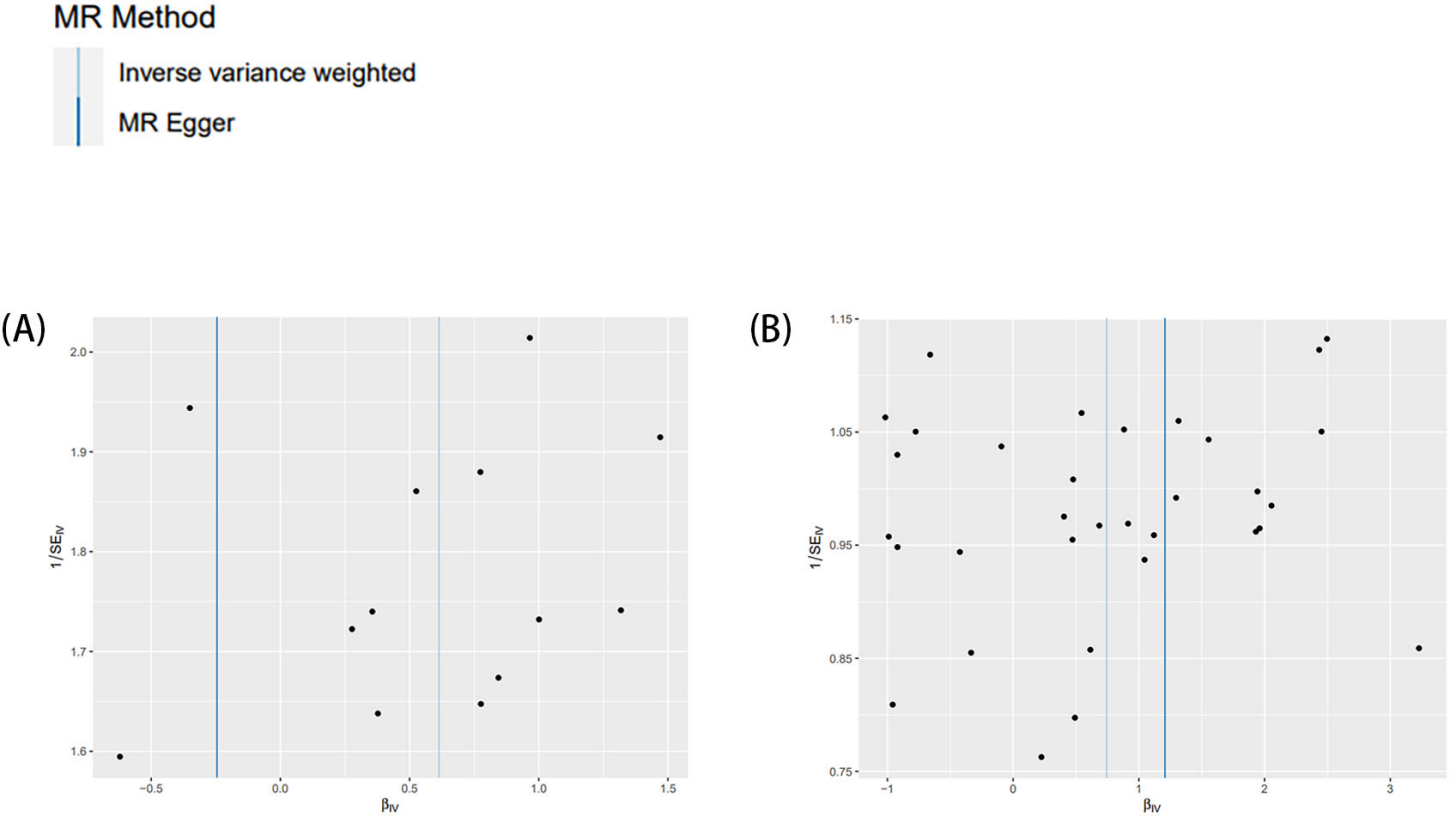
Funnel plots employed to assess heterogeneity. Exposure two screen times and outcome ADHD. **(A)** length of mobile phone use; **(B)** time spent watching television.

**Figure 4:**
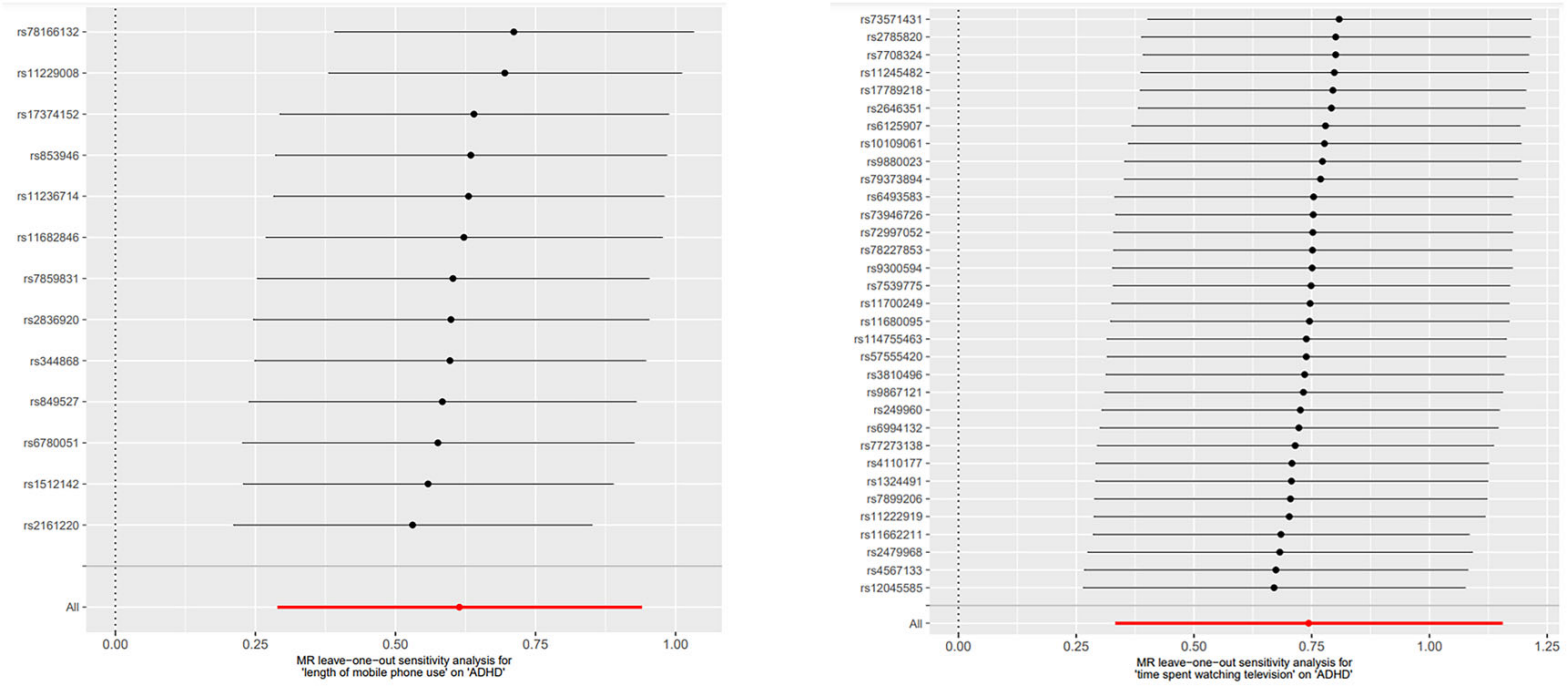
Leave-one-out diagrams of MR analyses. Exposure two screen times and outcome ADHD.

The MR-Egger regression intercept results indicated the absence of horizontal pleiotropy in all MR analyses (**Table 4**). In the MR analyses, heterogeneity was observed solely for the time spent using computer, suggesting the presence of confounders in the SNPs used for MR analyses (**Table 4**). However, as the MR analysis results for this category of screen time and childhood ADHD were not statistically significant, they did not impact the conclusions drawn. The funnel plot’s symmetry also led to the same conclusion (**Figure 3**). Leave-one-out sensitivity analyses demonstrated that no one SNP was responsible for the causal relationships between two types of screen time (the length of mobile phone use and the time spent watching television) and childhood ADHD (**Figure 4**).

**Table 4:** Pleiotropy and heterogeneity test of screen times in ADHD GWAS.

Exposure	Outcome	Pleiotropy test		Heterogeneity test			
		MR-Egger intercept	p	IVW		MR-Egger	
				Cochran's Q	p	Cochran's Q	p
Length of mobile phone use	ADHD	0.018	0.203	13.558	0.330	11.625	0.393
Time spent using computer	ADHD	-0.009	0.492	60.953	0.002	60.045	0.002
Time spent watching television	ADHD	-0.006	0.558	44.657	0.068	44.157	0.059

## 4 Discussion

This study utilized bidirectional two-sample MR analysis to evaluate the genetic underpinnings of the causal relationship between screen time and childhood ADHD. From the available large-scale GWAS pooled data, four categories of data characterizing screen time were extracted. The findings of this study indicated that the time spent watching television and the length of mobile phone use was significant causally associated with the disorder only in IVW and Weighted median method. This indicates that the causality we have identified may not be as robust as observed and warrants further investigation through more comprehensive and detailed studies.

The rising prevalence of ADHD among children has significant financial implications for societal development, family relationships, and personal advancement. As the prevalence of electronic device usage among children and adolescents continues to increase, it is imperative to assess the impact of screen time on their physical health, cognitive development, and overall growth. Previous research has demonstrated a growing interest in exploring the correlation between excessive screen exposure and adverse effects on various aspects of health, including psychological, social, and neurological effects (10). Meanwhile, Observational studies have indicated that prolonged screen time may have detrimental consequences on mental and physical health outcomes, such as obesity and myopia, in the pediatric population. A cohort study involving 3826 adolescents suggests that the use of social media and television may exacerbate depressive symptoms (19). A meta-analysis further indicates a correlation between screen time based on sedentary behavior and an elevated risk of depression (20). A review covers studies from 1999 to 2014 revealed that the majority of previous studies had found negative associations between screen usage and both shorter sleep duration and increased sleep difficulties (21). A cross-sectional Canadian study suggests that screen use may serve as a risk factor for anxiety and sadness among teenagers (22). Excessive screen time is related to a greater incidence of depression among adolescents and heightened levels of anxiety in both males and females. Furthermore, increased exposure to screen media has been linked to a greater risk of adolescent obesity (23). Prolonged periods of sedentary behavior in front of a screen are strongly correlated with negative health outcomes, such as suboptimal cognitive-behavioral functioning and diminished psychosocial wellbeing.

Excessive screen usage can affect the prevalence of ADHD at the behavioral level through visual or auditory stimuli. When presenting with stimuli unrelated to behavioral reactions, those with ADHD exhibit increased brain activity (24). Research indicates that a fast-paced program of screen media may exacerbate ADHD-related behaviors, including attentional difficulties in children (25). An experimental study utilizing a mouse model of excessive sensory stimulation corroborated that prolonged exposure to audiovisual media during childhood results in cognitive and behavioral deficits (26). Similarly, longitudinal naturalistic studies have demonstrated that early screen exposure is associated with diminished executive functioning in children (27). Research has demonstrated that children with ADHD engage in screen-based activities to meet their reward sensitivity needs (28), which results in them using screens more frequently than normal kids. Screen-based activities may decrease children’s use of motor functions and increase their likelihood of exhibiting symptoms of ADHD. According to another research, eye movement-focused interventions have also been found to improve cognitive and motor functions in children with ADHD (29). Additionally, children who engage in screen-based activities may be less likely to use their kinesthetic senses, which could predispose them to exhibit symptoms of ADHD. Furthermore, numerous studies have demonstrated that physical activity can enhance executive functioning in children diagnosed with ADHD (30).

Screen time may have an indirect impact on childhood ADHD by altering various lifestyle factors. Specifically, the severity of ADHD symptoms in children may be influenced by increased screen time, which can lead to decreased sleep quality or exacerbate existing sleep disturbances. A study involving 374 French children found that nighttime screen usage significantly affected sleep disruptions, which in turn were directly associated with symptoms of ADHD in this population (31). A study conducted in Shanghai, China found that screen usage before bedtime exacerbated symptoms of ADHD in children (32). Additionally, teenagers with ADHD who use screens at night experience shorter sleep durations and increased sleep disturbances (33). Studies conducted in English-speaking countries have indicated that lifestyle factors such as screen time may play a mediating role in the association between childhood ADHD and sleep quality (34). Furthermore, a mediation analysis involving nearly 4,000 high school students in Canada identified impulsivity as a significant mediator of ADHD symptoms among underlying behaviors brought on by screen usage (35). Therefore, we can employ mediated Mendelian randomization for further causal interpretation in subsequent studies to provide additional insight into the causal mechanisms underpinning the effect of screen time on childhood ADHD.

This study examined the genetic basis of the causal association between screen time and childhood ADHD through four perspectives and utilized five different models to assess causality for the first time. The potential cause-and-effect relationship between childhood ADHD and screen time highlights the importance of implementing effective screen time control measures for children and adolescents. Nevertheless, this study has some limitations. Firstly, it should be noted that not all employed methods substantiated the causal relationship we identified. Secondly, through examining scatter plots, we observed that the statistically significant causality might be dominated by certain SNPs, which could be pivotal in the association between screen time and childhood ADHD. Therefore, it is crucial to have access to larger data samples and employ more advanced MR analysis methods to obtain more accurate and precise relationships.

## 5 Conclusion

This study investigated the underlying causality between screen time and childhood ADHD through a genetic lens. The findings suggested that screen time may be a significant factor in the formation and development of ADHD in children, providing novel insights for further investigation into preventive measures for ADHD in this population.

## Data Availability

Publicly available datasets were analyzed in this study. This data can be found here: https://gwas.mrcieu.ac.uk/datasets/ukb-b-4094/; https://gwas.mrcieu.ac.uk/datasets/ukb-b-17999/; https://gwas.mrcieu.ac.uk/datasets/ukb-b-4522/; https://gwas.mrcieu.ac.uk/datasets/ukb-b-5192/; https://figshare.com/articles/dataset/adhd2022/22564390.

## Footnotes

1 Qu Y, Huang X, Mi J, Chen X, Wu B, Xu J. Education, socioeconomic status, leisure sedentary behaviours and female infertility: Mendelian randomization study. Research Square. Under Review Jan 08, 2024. doi: 10.21203/rs.3.rs-3770062/v1
